# Serious gaming and eye-tracking for the screening, monitoring, and diagnosis of neurodevelopmental disorders in children: a systematic literature review

**DOI:** 10.3389/fbioe.2025.1672718

**Published:** 2026-01-14

**Authors:** Muhammad Farooq Shaikh, Ciara Higley, Cecilia Campanile, Rebecca Francis, Elyssa Panja, Silvia Santacaterina, Giacomo Pratesi, Davide Piaggio

**Affiliations:** 1 Applied Biomedical Signal Processing Intelligent eHealth Lab, School of Engineering, University of Warwick, Coventry, United Kingdom; 2 The Beacon Academy Internship, Warwick Medical School, University of Warwick, Coventry, United Kingdom; 3 Paperbox Health S.r.l, Torino, Italy; 4 Unità Operativa Complessa di Otorinolaringoiatria (U.O.C), Fondazione Policlinico Universitario Campus Biomedico di Roma, Roma, Italy

**Keywords:** neurodevelopmental disorder, learning disorder, serious gaming, ADHD, dyslexia, mHealth, digital health

## Abstract

Neurological development between the ages of 3–11 is crucial to the shaping of infrastructural capabilities like the executive functions that enable the child to achieve academically and socially. Such development can be hindered by neurodevelopmental disorders (NDDs) like attention deficit hyperactivity disorder (ADHD), Dyslexia, and Dysgraphia, which affect 5%–10% of the world population of children. Although the importance of early screening is acknowledged, inadequacies such as access barriers, long waiting time, and excessive cost lead to late detection, even when potential issues are identified. This PRISMA-based systematic review examines the role of technology and serious games that may screen and monitor NDDs in children early. The PubMed and Scopus databases were utilized, and research published between 2013, and February of 2025 was reviewed, where the age interval of the sampled children was between 3 and 11, and extended to 21 in relevant cases. Some of the tools reviewed are eye-tracking systems, machine learning models, mobile applications, and serious games. The quality of studies was assessed by the Mixed Methods Appraisal Tool (MMAT) and the results synthesized narratively. Out of 3,129 records, 37 studies were included according to the inclusion criteria. Findings indicated that although numerous technologies showed promise in recognizing and assisting children with NDDs, the majority had limited capabilities in scalability, longitudinal tracking, and practical application as the following was minimal, and the length of follow-up was low. In summary, the possibilities of using technology to better diagnose and intervene early are promising, although cost, training and implementation frameworks aligned with the NHS are critical barriers.

## 1 Introduction

Neurodevelopmental disorders are a group of disorders causing deficits in cognition and delayed brain development due to various causes, including dysregulation of mechanisms governing the development of the cerebral cortex (Damianidou et al., 2022). Currently, it is reported that 5%–11% of children under 18 have been diagnosed with attention-deficit/hyperactivity disorder (ADHD), 3%–10% with a specific learning disorder (SLD), and 0.63% with an intellectual disability. There is limited data available from low-income countries on neurodevelopmental disorders (Hadders-Algra, 2021), which accounts for a discrepancy between the accurate prevalence of neurodevelopmental disorders (Frances et al., 2022; American Psychiatric Association, 2013).

The main neurodevelopmental disorders we are focusing on are dyslexia, dyscalculia, dysgraphia, which can be classed as SLD, a subset of neurodevelopmental disorders, and ADHD. The Diagnostic and Statistical Manual of Mental Illnesses, fifth edition (DSM-5) classifies dyslexia as the inability to link word patterns and pronunciations, affecting reading ability both internally and externally (Snowling and Hulme, 2012). People with dyscalculia struggle with ‘numeric and arithmetic’ concepts (Castaldi et al., 2020), whilst those with dysgraphia have lower abilities in fine motor coordination, than neurotypical individuals (Chung et al., 2020). Finally, ADHD is diagnosed when an individual exhibits reduced attentiveness and hyperactivity with actions of impulsivity (Braithwaite et al., 2020).

Neurodevelopmental disorders are costly to both individuals and society. Disability adjusted life years (DALYs) are a quantification of years of full health lost to an individual. The DALYs associated with ADHD are 13.78 (Jepsen and Younossi, 2021), and data suggest that the quality of life for individuals suffering from dyslexia, dyscalculia and dysgraphia was significantly lower in several domains than neurotypical subjects (FragaGonzlez et al., 2018; Hen-Herbst and Rosenblum, 2022; Reddy and Reddy, 2016). Individuals suffering from a learning disorder are twofold less likely to gain a degree qualification after a full education. Such individuals are also more likely to be unemployed than neurotypical peers (Aro et al., 2019; Baig et al., 2019). In addition to this, poor mental health, emotional and behavioral issues are associated with specific learning disorders (Sahoo et al., 2015). The overall cost to an individual due to the burden of learning disorders can be categorized into medical (for diagnosis, treatment, and rehabilitation), non-medical (transport to appointments) and indirect costs (productivity losses, loss of earnings and academic enhancement measures). In 2012, the average annual cost to an individual with ADHD was estimated to be between £8,422.68 and £12,371.77, however, as more individuals are being diagnosed with multiple neurodevelopmental disorders, this burden is increasing (Kularatna et al., 2022). The direct, indirect, and intangible costs due to SLD in India were INR 5.94M, 29.26M, and 42.30M, respectively, with indirect costs making up 83.1%. Recent estimates show annual costs per child of £97,823.09 in India and total national expenditure of £569.48M in Australia (Karande et al., 2019; Arora et al., 2020).

According to the current guidelines of the National Institute of Health and Care Excellence in England (UK NICE) diagnosis of ADHD includes a clinical evaluation of behavioral symptoms based on standard rating scales, structured interviews, and multi-informant reports, which show high inter-rater reliability across international diagnostic systems (Tannock, 2013). There are dedicated methods such as the Strengths and Difficulties questionnaire or Conners’ rating scale (NICE, 2019). Conners’ rating scale is for the teacher, parent, and child self-report of conduct, self-care, and academic performance, and literacy level. SLD can be diagnosed by assessing intelligence, memory, and other cognitive skills of a child for basic reading, writing, and counting abilities using this rating scale (Mather and Schneider, 2023). These assessments are currently carried out by an educational psychologist or a neurodevelopmental healthcare professional (Roberts et al., 2012). Access to diagnostic and therapeutic services varies globally; in higher-income countries, shortages of trained professionals often cause long waiting times and delayed diagnoses, whereas in lower-income regions, cultural stigma and limited mental health awareness remain major barriers to seeking care (Kieling et al., 2011). Early screening is indeed paramount, as it allows treatment to begin earlier, allowing the individual to receive support from an early age and reducing the costs to the individual and society (Sonuga-Barke et al., 2011). The current most effective treatment for ADHD is stimulants (methylphenidate and amphetamine) alongside behavioral therapy, boosting both dopamine and noradrenaline levels (Nazarova et al., 2022). However, non-stimulant medications such as atomoxetine and guanfacine have also demonstrated comparable efficacy among responders, particularly in cases with stimulant intolerance or comorbid conditions (Kratochvil et al., 2003). Currently, there are no medications that directly treat dyslexia, dysgraphia, or dyscalculia. However, in comorbid cases involving ADHD, non-stimulant medications such as atomoxetine have shown secondary improvements in reading attention and cognitive control, indirectly supporting literacy-related outcomes (Kratochvil et al., 2003; Verwimp et al., 2024).

From the technological advances point of view, in recent years, it has been proposed that ‘serious gaming’ and ‘visual training’ can be used for the diagnosis and treatment of several learning disorders (e.g., dyslexia) (Khaleghi et al., 2022; Bucci, 2021). Similarly, eye tracking has been identified as a potential diagnostics and rehabilitation tool in both ADHD (Stokes et al., 2022; Lee et al., 2021) and dyslexia (Vajs et al., 2023; Jafarlou et al., 2017). In fact, eye-tracking technology delivers exceptional assessment abilities for neurodevelopmental disorders when its measurements exist independently from other measurement procedures. The objective and quantifiable capability of eye-tracking technology allows users to measure direct visual attention through oculomotor control to gauge gaze patterns, which commonly surface in ADHD and autism spectrum disorder as well as dyslexia patients. Furthermore, this technology allows clinicians to view subtle signs of abnormal thinking and vision processes which standard ways of testing might overlook (Takahashi et al., 2024; Fernández-Martín et al., 2024). Thus, early diagnostic symptoms may become evident. Moreover, eye-tracking monitoring now exists as a component within low-cost non-invasive screening arrangements, which allow its utilization in schools for early diagnosis and remote care. It also helps in rehabilitation by regularly watching how treatment is progressing. Additionally, these diagnostic capabilities of eye-tracking offer high accessibility along with scalability because the system provides real-time data while avoiding any need for verbal communication (Yoo et al., 2024; Gomolka et al., 2024). The analysis of eye movements has proven its standalone diagnostic capacity through reliable research findings indicating predictive and classification benefits that appear when using machine learning approaches or standalone measures (Oh et al., 2024). Research analysis of eye-tracking requires its own assessment because of its worthiness for presentation in this review (Toki et al., 2024).

Hence, this systematic literature review examines the available literature on the use of serious gaming and eye-tracking technologies for the early screening, diagnosis, and monitoring of the aforementioned neurodevelopmental disorders in children. This systematic literature review is carefully restricted to the most studied neurodevelopmental and SLDs in children, including ADHD, dyslexia, dysgraphia, dyscalculia and general reading difficulties. The research results will help guide the creation of a clinical decision support system for such disorders that leverages AI and consumer-grade electronics (e.g., smartphones and tablets) to assist Paperbox Health’s endeavors. Paperbox Health is a start-up based in Italy that specializes in the detection and treatment of neurodevelopmental disorders using serious gaming.

## 2 Methods

**FIGURE 1:**
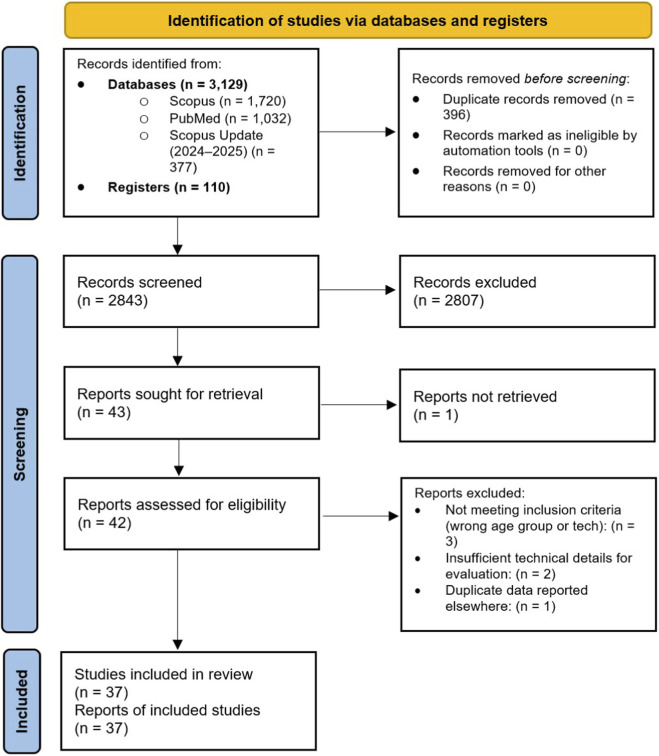
PRISMA flow diagram illustrating the screening and selection process for included studies.

The methodology followed the PRISMA statement for systematic literature reviews, ensuring a structured and transparent approach to reporting (see Figure 1) (Yepes-Nuez et al., 2021).

The completed PRISMA checklist has been provided as Supplementary Material 1 to confirm adherence to established guidelines and facilitate reproducibility (Yepes-Nuez et al., 2021).

## 3 Search strategy

To perform this systematic review, a detailed and reproducible search strategy was developed. Meaningful keywords were identified through expert input and review of relevant literature. PubMed MeSH terms were used to structure part of the query and identify relevant synonyms. A focus group consisting of experts in biomedical engineering, medicine and speech and language therapy (SALT) finalized the search string. Boolean logical conjunctions (AND) along with disjunctions (OR) functioned to link search terms among five established categories such as neurodevelopmental diseases, digital gaming platforms, computational learning systems, child participants and assessment systems. All search strings employed for PubMed and Scopus investigation can be found in Supplementary Material 2. These search strings were inputted into Scopus and PUBMED, identifying research papers and possible clinical trials from January 2013 to February 2025. PubMed and Scopus were chosen as the most extensive and universal biomedical and interdisciplinary databases, and provided a broad range of engineering, medical and behavioral science research on the topic (Molina et al., 2016). Finally, one last search was performed on specific registries of clinical trials, specifically clinicaltrials.gov, using the same keywords to ensure capturing also this kind of evidence.

## 4 Inclusion/exclusion criteria

After assessing existing reviews, we decided to continue with our systematic review effort, as we did not find any similar effort in the recent literature. We included only journal articles published in English from January 2013 to February 2025, related to screening neurodevelopmental disorders in children, possibly the studies that included children up to the age of 18 were included. The English language requirement was introduced as English is the only language common to all authors as well as because most of the evidence is, in any case, published in English (Hyland, 2016).

Also studies with children already diagnosed with learning disorders were included, undergoing a technology-based intervention for the screening of neurodevelopmental disorders. Although the inclusion criteria were made to include studies in which children up to the age of 18 years were used, some studies were also included in which the upper age limit was slightly expanded (e.g., up to 21 years). These exceptions were allowed when the study samples mainly included school-aged children and when the results of the studies had a direct impact on the objectives of the review on screening, monitoring, or diagnostic technologies. We excluded reviews, conference papers, book chapters, editorials, notes, and those focusing only on epidemiology or using electroencephalography (EEG) or functional magnetic resonance imaging (fMRI) (see Table 1). Two authors independently appraised articles by title, abstract, and full text, with a third-party resolving discrepancy, if needed.

**TABLE 1:** Inclusion and exclusion criteria used in the systematic literature review.

Inclusion criteria	Exclusion criteria
Publications from January 2013 to February 2025	​
Original articles only	No conference papers, book chapters, reviews, editorials
Studies that included children up to the age of 18 were included. Also studies with children already diagnosed with learning disorders were included	Studies did not include any children up to the age of 18
English language	Any other language
Any study or manuscript describing how serious games, eye-tracking, machine learning or smartphone/tablet applications are applied for the screening, diagnosis, and monitoring of the aforementioned neurodevelopmental disorders	No use of electroencephalography (EEG) or functional magnetic resonance imaging (fMRI), no robots

## 5 Data extraction and quality appraisal

Relevant data was extracted and collated into an ad-hoc Excel spreadsheet. The quality appraisal was conducted using the MMAT tool (Hong et al., 2018), as the finalized selection included multiple study types. The MMAT tool allows for analysis of quantitative and qualitative studies.

## 6 Data synthesis

The narrative synthesis method (Booth et al., 2025) was used for data synthesis of the extracted data. For each paper, the type of disorder, technology and overall aims and conclusions of the study were identified and organised by the type of neurodevelopmental disorder. This information was used to generate discussions and future directions.

## 7 Results

### 7.1 Search outcome

This systematic literature review integrated PubMed and Scopus databases for data retrieval together with clinicaltrials.gov. The combined search returned 3,239 records (see Figure 1). After duplicate removal (396), the total number of screened records reached 2,843. After title and abstract screening, only 43 records seemed to be in line with the inclusion and exclusion criteria. Finally, a thorough full-text evaluation led us to the final set of 37 studies to be included in this review.

### 7.2 Data visualization and extraction

Table 2 contains the summary of the data extracted from the included studies. The following sections will report the findings by type of disorder and technology used.

**TABLE 2:** Summary of data extracted from the included studies, detailing the types of neurodevelopmental disorders investigated, the technologies employed in the research, the aims of each study, the number of participants and their age ranges, and the reported outcomes.

Article No citation	Type of disorder	Technology	Aim of the study	Participants	Conclusion of the study
Apiquian et al. (2020)	ADHD	Video game	To determine whether their video game (Chefmania) was a useful tool in screening ADHD	266 participants, aged 6–12	The intervention, consisting of Chefmania scores, showed that children with ADHD, aged 6–12 performed poorly, suggesting the potential of Chefmania as a valid tool for assessing cognition in schools
Fernández-Martín et al. (2024)	ADHD	Educational video games	To explore modern techniques which can be used to create data models for ADHD based on ecological assessments of attention problems and distractibility combined with hyperactivity indicators, using virtual reality	110 participants, with a mean age of 9.47 (standard deviation of 2.93)	The research produced distinctive behavioral profiles for improving the delivery of ADHD interventions
García-Baos et al. (2019)	ADHD	Eye-tracking video game	To assess the therapeutic potential of an eye-tracking game (RECOGNeyes) for training visual attention and oculomotor control in children with ADHD	28 participants, aged 8–15, previously diagnosed with ADHD (DSM-5 criteria)	The eye-tracking group showed significant improvements in reaction time, gaze fixation, and impulsivity control, suggesting RECOGNeyes may be a useful non-pharmacologic tool for ADHD training
Garcia-Redondo et al., 2019)	ADHD	Educational video games	To analyze the effects of a serious game based on multiple intelligences on attention in students with ADHD and SLD, using performance and observation measures	44 students, aged 6–16	The intervention, consisting of 28 sessions using educational video games, resulted in a significant improvement in attention performance, particularly visual attention, suggesting the potential of serious games in addressing attention issues in students with learning disabilities
Garcia-Zapirain et al. (2017)	ADHD	Eye-tracker and hand gesture sensors	To evaluate a rehabilitation system using physiological sensors	19 participants, aged 10	The system, based on user feedback, showed high effectiveness in rehabilitation, suggesting its potential in improving rehabilitation outcomes
Heller et al. (2013)	ADHD	Machine learning, video game	To test the accuracy of a game implementing machine learning at identifying ADHD.	52 participants, aged 6–17	The game was able to accurately detect combined type ADHD 75% of the time and inattentive type 78% of the time demonstrating the potential to create a game to detect patterns for ADHD and potentially other mental disorders
Oh et al. (2024)	ADHD	Eye-tracking	The research investigates the effectiveness of AttnKare-D, an artificial intelligence software that evaluates Attention Disorders using virtual reality evaluations	20 children aged 6–12 (15 ADHD, 5 control)	Virtual Reality (VR) and AI integration improved diagnostic accuracy, simulating real-life environments
Slobodin et al. (2020)	ADHD	Data collection using MOXO-CPT	To use a machine-based learning model to predict ADHD in children	458 participants, aged 6–12	The MOXO-CPT assessment predicted ADHD with high accuracy, quicker and more cost effective than the current ADHD diagnosis showing 87% accuracy, 89% sensitivity and 84% specificity
Takahashi et al. (2024)	ADHD	3D video game, and eye-tracking	To investigate if ADHD children can be assessed through executive function performance on a 3D action puzzle video game	33 participants aged 8–21	The game metrics showed parallel results to neuropsychological test results which suggest that the game can evaluate executive functioning in patients with ADHD.
Toki et al. (2024)	ADHD, neurological disorders	Educational video games	To explore machine learning methods to forecast possible neurodevelopmental disorders among children	473 participants for the model; 184 participants with an average age of 7 for the predictive analysis	ML models demonstrated good prediction capabilities for early diagnosis, based on cognitive features
Türkan et al. (2016)	ADHD	Video-based eye-tracking	To investigate oculomotor abnormalities in ADHD	48 participants (24 children with ADHD and 24 typically developing children, mean age = 8 years, 10 months)	The intervention showed that children with ADHD, aged 6–17, had shorter fixation periods compared to neurotypical peers, suggesting differences in attention performance
Yoo et al. (2024)	ADHD	Eye-tracking	To explore the creation of an eye-tracking feature-based machine learning screening system for ADHD.	135 participants (56 with ADHD and 79 typically developing children), with an average age of 8.6 years old	The research team achieved 76.3% accuracy using eye-tracking-only features, which demonstrates the potential of ML models for ADHD screening
Sweere et al. (2022)	ADHD, neurological disorders	Eye-tracking and manual response	To investigate distraction effects during reaction tasks	177 participants, aged 6–17	The intervention, based on eye movement data, demonstrated increased distractibility in ADHD/neurological groups, with no significant latency differences, suggesting potential insights into attention performance in these groups
Perochon et al. (2023)	Autism and ADHD	Tablet based game	To use extracted touch features from a bubble popping game to differentiate between neurotypical and neurodivergent children	233 participants, aged 1.5–10	The intervention, involving touch-based games, showed that autistic children with co-existing ADHD took longer to pop the bubble and had a longer touch length on average, suggesting the potential of touch-based games as an efficient approach to screen autism and ADHD.
Van Viersen et al. (2013)	Dyscalculia	Eye-tracking	To examine abnormal estimation patterns in dyscalculia	11 participants (a 9-year-old girl with developmental dyscalculia and 10 typically developing children)	The intervention, using eye-tracking, effectively identified abnormal estimation patterns in dyscalculia, suggesting its potential as a tool for diagnosing dyscalculia
Piazzalunga et al. (2023)	Dysgraphia	Visual perception games, and eye-tracking device	To investigate the role of visual perception in handwriting skills	53 participants, aged approx. 8	The intervention showed that children with dysgraphia displayed more inattentiveness and indecisiveness, suggesting the potential of this tool to predict risks in handwriting with good accuracy
Alt et al. (2017)	Dyslexia	Computer-based word learning games	To use word learning games for identifying strengths and weaknesses in children with dyslexia by measuring their ability to link names and objects. Analyses of covariance and nonverbal intelligence scores assessed phonological-visual linking, mispronunciation detection, naming, visual difference decision, and visual feature recall	184 participants, aged 7–8	The study found word learning deficits in children with dyslexia across all manipulations and most task-manipulation combinations, especially in phonological tasks. Visuospatial manipulations showed mixed effects
Gomolka et al. (2024)	Dyslexia	Eye-tracking	To diagnose dyslexia in early school-aged children using eye-tracking and LSTM networks	145 children aged 7–10	The Model displayed 97.7% accuracy making it suitable for diagnosing dyslexia in its early stages
Ikermane and El Mouatasim (2023)	Dyslexia	Webcam-based eye-tracking	To study reading behavior using Arabic text configurations	61 participants, aged 9–13	The intervention, using webcam-based eye-tracking, showed that dyslexic individuals exhibited longer fixations and reading periods, suggesting its diagnostic potential for dyslexia
Jafarlou et al. (2017)	Dyslexia	Videonystagmography (VNG)	To assess oculomotor rehabilitation in dyslexia	50 participants aged 8–12 years (30 dyslexic children and 20 typical readers)	The intervention, involving oculomotor rehabilitation, improved eye movement patterns, suggesting its potential to aid reading and visual search abilities
Jafarlou et al. (2021)	Dyslexia	Eye-movement tracking	To evaluate the effectiveness of oculomotor rehabilitation on the cognitive performance of dyslexic children	50 participants aged 8–12 years (30 dyslexic children and 20 non-dyslexic children)	The intervention showed a significant difference between children with and without dyslexia in signs of oculomotor impairment, suggesting that early diagnosis of eye movement disabilities can improve cognitive performance in affected children
Korneev et al. (2018)	Dyslexia	EyeLink 1000 eye-tracking device	To investigate reading mechanisms in young readers	36 participants, aged approx. 9	The intervention showed that longer words caused more fixations, suggesting that proficient readers used lexical strategies more effectively
Larco et al. (2021)	Dyslexia	Web and app-based learning game	To create web and mobile apps to provide learning resources to dyslexic children aged 7–10 years	25 participants, aged 7–10	The intervention, involving the ‘Helpdy’ app, showed improvement in the symptoms of dyslexia in children, suggesting its potential as a tool for supporting dyslexia management
Masulli et al. (2018)	Dyslexia	Video-based eye-tracking	To investigate effects of text format on eye movements	45 participants aged 7–12 years (15 dyslexic, 15 reading-age-matched non-dyslexic, and 15 chronological-age-matched non-dyslexic)	The intervention showed that dyslexic children had longer fixation durations, with font size and spacing reducing fixation durations, suggesting their potential in improving reading performance for dyslexic individuals
Moiroud et al. (2018)	Dyslexia	Eye-tracking with DEM Test	To analyze oculomotor behavior in dyslexic children	39 participants aged 7–12 years (13 dyslexic, 13 chronological-age-matched non-dyslexic, and 13 reading-age-matched non-dyslexic)	The intervention showed that dyslexic children had longer fixation durations, with font size and spacing reducing fixation durations, suggesting their potential in improving reading performance for dyslexic individuals
Nilsson et al. (2016)	Dyslexia	Video-based eye-tracking	To predict dyslexia risk using eye-tracking data	185 participants aged 9–10 years (97 high-risk with early word decoding difficulties and 88 low-risk control)	The intervention, using eye-tracking, predicted dyslexia risk with 96% accuracy, suggesting its potential as a reliable tool for early dyslexia detection
Prabha and Bhargavi (2020)	Dyslexia	Machine learning with eye-tracking	To compare dyslexic vs. non-dyslexic traits using ML models	187 children aged 9–10 years old, of which 97 were dyslexic and 88 non-dyslexics	The PSO-SVM Hybrid Kernel achieved 95.6% accuracy in identifying dyslexia traits, suggesting its potential as an effective tool for dyslexia detection
Jothi Prabha and Bhargavi (2022)	Dyslexia	Machine learning with eye-tracking	To develop screening tools for dyslexia diagnostics	187 children aged 9–10 years old, of which 97 were dyslexic and 88 non-dyslexics	The intervention validated the PSO-SVM Hybrid Kernel as an effective screening tool for schools, suggesting its potential in identifying dyslexia traits
Rauschenberger et al. (2022)	Dyslexia	Web-game	To develop a game for universal screening of dyslexia	313 participants aged 5–10 years (116 with dyslexia, 197 non-dyslexic)	The intervention showed seven separate variables with significant differences between those with and without dyslexia, suggesting that this approach can optimize resources for detecting dyslexia, though it needs to expand training data
Rello et al. (2020)	Dyslexia	Online gamified test	To design an online gamified test and a predictive machine learning model for dyslexia detection in Spanish speakers	3,644 participants (general age group unspecified) and 1,300 participants aged 12 years or older in a follow-up study	The study correctly detected 80% of participants with dyslexia among over 3,600 participants, suggesting the potential of the online screening tool, used by over 200,000 people, for early detection and prevention of dyslexia-related challenges
Vajs et al. (2023)	Dyslexia	Remote eye-tracking (SMI RED-m)	To analyze eye movements while reading with color configurations	30 participants aged (15 dyslexic and 15 neurotypical)	The intervention, using eye-tracking data, effectively differentiated dyslexic and control groups with high accuracy, suggesting its potential as a reliable tool for dyslexia detection
Vajs et al. (2022)	Dyslexia	Spatiotemporal eye-tracking	To differentiate dyslexic and non-dyslexic readers	30 participants aged 7–13 years (15 dyslexic and 15 non-dyslexic)	The intervention, using spatiotemporal features, achieved 94% accuracy in distinguishing dyslexic readers, suggesting its potential as an effective tool for dyslexia identification
van de Ven et al. (2017)	Dyslexia	Reading game (letter prince)	To measure the impact of a multicomponent reading game on the development of reading skills and motivation in 60 first grade Dutch schoolchildren with special educational needs	60 participants aged primary school age (Dutch children with special educational needs)	The short intervention (9 × 15 min) improved pseudoword and text reading fluency, with early intervention reducing reading time and late intervention increasing pseudoword identification, showing no impact on reading motivation
Bonifacci et al. (2023)	Dyslexia, preterm birth	Video-based eye-tracking	To compare eye movements in dyslexia and preterm birth	78 participants, aged 10.5 years (preterm, dyslexic, and typically developing children)	The intervention showed that dyslexic children had increased fixations and smaller saccades, while preterm children exhibited reduced reading speed, suggesting potential differences in reading performance
Schmitt et al. (2018)	N/A	Web-based educational video games	To evaluate the effectiveness of an educational website in promoting the literacy skills of young children via a randomized trial over 8 weeks (about 2 months)	136 participants, aged 4–7	The intervention, using the website, showed that children had better literacy results after assessment compared to the control group, suggesting its potential to improve literacy outcomes
Peters et al. (2020)	Reading difficulties	Eye-tracking	To examine the relationship between RAN and reading skills	93 participants, aged 5–8	The intervention, using fixation metrics, predicted RAN performance and differentiated between reading difficulties and neurotypical peers, suggesting its potential for identifying reading challenges
Niemela (2020)	Reading disability	Serious Games and clustering	To implement a new clustering-based approach for identifying different profiles of serious	136 participants, with varying age (specific age not mentioned), all learners of GraphoLearn analyzed using a clustering-based profiling method to detect reading difficulties	The intervention showed that it is possible to identify different types of learners, suggesting its potential for tailoring educational approaches

### 7.3 Dyslexia

Dyslexia was the most extensively studied disorder, featuring 18/37 (48.6%) of the included studies, with technologies such as eye-tracking, educational games, and machine-learning-based software receiving significant attention.

Eye-tracking was a particularly prominent tool, offering insights into the reading behaviors and oculomotor patterns of individuals with dyslexia. For instance, Vajs et al. (2023), Vajs et al. (2022) demonstrated that spatiotemporal features (e.g., fixation intersection coefficient and fixation fractal dimension) extracted through eye-tracking could distinguish dyslexic from non-dyslexic readers with 94% accuracy, based on a group of 30 children. Similarly, Vajs et al. (2023), ilsson et al. (2016), Vajs et al. (2022). Achieved 96% accuracy in predicting dyslexia risk using video-based eye-tracking, based on 97 high-risk subjects and a control group of 88 low-risk subjects, while Korneev et al. (2018) investigated the reading mechanisms of 36 second-grade pupils using eye tracking. Their results highlighted a close relation between the level of the reading skill and the oculomotor activity. Additionally, Bonifacci et al. (2023) utilized eye-tracking to compare reading behaviors among children with dyslexia, those with typical development, and those born preterm, identifying distinct oculomotor patterns in both groups, such as more fixations and more frequent and smaller saccades for the groups affected by dyslexia. Prabha and Bhargavi (2020) focused on eye-tracking as a means of analyzing cognitive processing in dyslexics using machine learning models to compare dyslexic versus non-dyslexic traits. Specifically, fixations and saccades were detected and evaluated with machine learning algorithms. Gomolka et al. (2024), UCL (2013) implemented a new method for early dyslexia testing of school children through eye-tracking analysis by an LSTM-based deep learning model. The objective of the research focused on identifying dyslexic versus non-dyslexic children through oculomotor analysis of their reading tasks. Their research exhibited outstanding diagnostics outcomes, which confirmed the power of artificial intelligence models combined with eye-tracking technology for early dyslexia detection at educational institutions. Particle Swarm Optimization based SVM Hybrid Kernel was the best performing model, reaching 95.6% of accuracy. The same authors, in 2022, Jothi Prabha and Bhargavi (2022) conducted a subsequent study further validating the Particle Swarm Optimization based SVM Hybrid Kernel model on 187 kids, resulting in a proposed screening tool for the diagnosis of dyslexia in schools. Also, Masulli et al. (2018) studied eye movements, but aimed to compare the changes in eye movement between 15 children with and 15 children without dyslexia by reading four lines of text each with differing font sizes and word spacing. They found that increased font size and spacing caused a reduction in the duration of eye fixation and increased prosaccade amplitude in all children. They also found that the duration of fixation was significantly longer in dyslexic children. Jafarlou et al. (2017) evaluated the differences between eye movement patterns in Iranian dyslexic children compared to non-dyslexic children, with the latter presenting lower gain in pursuit and optokinetic tests, and increased latency with decreased accuracy in the saccade test. In their second study of 2021, they also assessed the effectiveness and impact of oculomotor rehabilitation on children with developmental dyslexia (Jafarlou et al., 2021). Moiroud et al. (2018) explored eye movement recordings during the Developmental Eye Movement test in dyslexic and non-dyslexic children. They found that children with dyslexia and non-dyslexic children of equivalent reading age have significantly longer fixation time and take longer to read than non-dyslexic children of similar chronological age. A significant correlation was also found between the fixation time and the number of words read in 1 minute with the total reading time. Similarly, Ikermane and El Mouatasim (2023) analyzed eye-tracking data of 61 subjects who were given Arabic normal text materials with various text configurations such as font-sizes, character spacing, and font family to analyse their reading behaviour. They found also that the eye motions of those with dyslexia differ from those of neurotypical readers. People with dyslexia tend to have longer and more frequent fixations and longer reading time. Results confirmed that webcam-based eye-tracking techniques can be used as an assessing tool for investigating the student’s reading performance and the potential risk of dyslexia. Finally, other innovative approaches included the use of different color configurations combined with eye-tracking. Vajs and Papi (2023) analysed the eye-tracking data from 15 dyslexic and 15 neurotypical Serbian school-age children who read 13 text segments presented on different color configurations (Vajs et al., 2023). They trained and tested a machine learning algorithm obtaining accuracies greater than 87%, and showing that the color configuration, despite it may influence each subject differently, does not affect the outcome.

Educational games were also widely explored as tools for both diagnosis and intervention. Rauschenberger et al. (2022) developed a web-based game (MusVis) which was able to observe significant differences between children with and without dyslexia, relying on seven significant variables including duration round, duration interaction and average click time. Larco et al. (2021) presented their app (Helpdy) which offers engaging educational games like *El Ahorcado* (Hangman) and *Memoria* (Memory) to enhance cognitive skills such as memory, concentration, and word recognition (Larco et al., 2021). The app also features two interactive pre-diagnosis games: one for phonological dyslexia (vowel placement to form words) and another for visual dyslexia (word selection tasks) (Larco et al., 2021). Rello et al. (2020) combined gamified online tests with machine learning models to predict dyslexia risk, achieving 80% success in Spanish-speaking populations, based on a study of more than 3,600 participants. Their game was designed based on typical mistakes made by Spanish people with dyslexia and on dyslexia-related indicators such as Language Skills, Working Memory, Executive Functions and Perceptual Processes (Rello et al., 2020).

Furthermore, van de Ven et al. (2017) proposed an adaptive learning platform driven by reinforcement learning to optimize reading practice for children with dyslexia, showing significant improvement in reading fluency (van de Ven et al., 2017). Specifically, they found a significant improvement in pseudoword naming and reading time after the use of a computer game. Alt et al. (2017) used word learning games and demonstrated that children with dyslexia face significant word learning deficits, especially when working memory demands are high, highlighting the interplay between cognitive load and language acquisition in dyslexic populations. However, the software they used was limited to a dichotomic choice (Yes VS No), with a 50% chance for the kid to guess the correct answer (Alt et al., 2017).

### 7.4 ADHD

ADHD was another well-represented disorder, featuring in 14/37 (37.8%) of the included studies, which explored video games, machine learning, eye-tracking, and physiological sensors as diagnostic and intervention tools. Several studies employed video games to assess cognitive abilities and attention performance. For example, Apiquian et al. (2020) used the “Chefmania” game assessing attention, memory, and impairment in executive functions to compare children with and without ADHD, showing a statistically significant difference, highlighting that this may be a plausible screening technique in this remit. They also noted that artificial intelligence could be used to allow adaptations for subjects with cognitive impairments (Apiquian et al., 2020). Similarly, Garcia-Redondo et al. (2019) demonstrated the potential of educational video games based on the Tree of Intelligences method (e.g., “Boogies Academy”) to improve attention in students with ADHD and specific learning disabilities, showing significant gains in visual attention after 28 sessions (Garcia-Redondo et al., 2019). This shows that computer intervention could not only act as a promising future diagnostic tool for ADHD but also offer a therapeutic advantage in the treatment of ADHD. Perochon (2023) introduced a tablet-based bubble popping game aimed at assessing visual-motor skills in children with ADHD and comorbid conditions such as autism. The study revealed distinct visual-motor patterns, including longer response times and higher error rates, indicating the potential of touch-sensitive games for early detection of ADHD-related symptoms in children also affected by autism (Perochon et al., 2023). Modern advancements in digital technology support several new studies that use them to improve ADHD detection. The assessment of executive functions in ADHD patients through 3D-video games was investigated by Takahashi et al. (2024), Schmitt et al. (2018). This research with 33 participants aged 8–21 demonstrated that in-game and executive functioning test results had a strong relationship which supports digital tools that mimic real-world environments for assessing ADHD.

Machine learning approaches were also applied to ADHD diagnostics. Heller et al. (2013) tested a game (“Groundskeeper”) integrating machine learning algorithms and demonstrated high accuracy in identifying ADHD subtypes, detecting combined-type ADHD with 75% accuracy and inattentive type with 78% accuracy. Similarly, Slobodin et al. (2020) employed a machine-based learning model within the MOXO-CPT framework inclusive of visual and auditory stimuli distractors, achieving 87% accuracy, 89% sensitivity, and 84% specificity in ADHD prediction.

Eye-tracking technologies provided additional insights into ADHD-related behaviors. Türkan et al. (2016) investigated oculomotor abnormalities in children with ADHD, identifying shorter fixation periods and higher distractibility compared to neurotypical peers. Sweere et al. (2022) further demonstrated how eye movement data from a visual reaction time task (“iDistrack”) can be used to quantify distractibility in ADHD populations, highlighting their heightened susceptibility to external stimuli. However, they also noted that the expected differences in manual press latencies between the ADHD and non-ADHD group were not observed (Sweere et al., 2022). Garcia-Baos et al. (2019) carried out a large-scale randomised control trial of interactive eye track game (RECOGNeyes) that aimed at training ocular motor control and visual attention in children with ADHD. During the study 28 participants were tested (ages 8–15) and the results proved that the participants who played the eye-controlled game performed better in terms of reaction time, fixation length of their gaze as well as being less impulsive as compared to the control groups who used mouse tracking. The results highlight the therapeutic effects of gaze-contingent video games in the improvement of visual attention systems and the oculomotor controls in ADHD groups of people (García-Baos et al., 2019). A portable eye-tracking system merged with machine learning algorithms became available for ADHD screening as described by Yoo et al. (2024), Niemela (2020). The research examined eye-tracking biomarkers among 56 participants aged 6–12 which showed movement latency and fixation time allowed researchers to achieve 76.3% accuracy in screening results. Studied results confirmed that cost-effective diagnostic systems employing eye-tracking technologies can work properly in medical environments. The research work of Oh et al. (2024), Vashishtha (2021) analyzed classroom simulations implemented in virtual reality with artificial intelligence elements during which 40 children aged 7–13 took part. The analysis through deep learning processing of their eye-tracking and behavioral activities showed early indications for real-world application success in ADHD prediction.

Other innovative tools included multimodal systems that combine various sensors and interfaces. For instance, Garcia-Zapirain et al. (2017) incorporated hand gesture recognition sensors and an eye tracker into a rehabilitation platform designed for ADHD intervention, receiving positive feedback on its usability and effectiveness in improving attention and motor coordination. A machine learning system by Toki et al. (2024), Forbes (2023) analyzed behavioral along with environmental data from 78 children between ages 6 and 12 to create the model. The research confirmed that AI models can make precise predictions about neurodevelopmental risks due to their general applicability. A virtual reality-based continuous performance test (AULA) helps Fernández-Martín et al. (2024), Peters et al. (2020) evaluated attention capabilities as well as hyperactivity and distractibility in 110 children between the ages of 6–16. New ADHD profiles emerged through cluster analysis which extended beyond DSM-5 subtypes because head motion and response time performances revealed themselves as effective clinical diagnosis tools.

### 7.5 Dyscalculia and dysgraphia

Dyscalculia and dysgraphia, although less frequently studied (2/37, 5.4%), have also shown potential for innovative technological interventions. While current data remains preliminary, there is compelling evidence to support the efficacy of these approaches. Research on dyscalculia has highlighted difficulties in spatial cognition and numerical representation, effectively identified through real-time eye-tracking analyses. For example, Van Viersen et al. (2013) employed eye-tracking to examine estimation patterns in individuals with dyscalculia, identifying distinct abnormalities in the processing of mathematical tasks. These findings underscore the utility of eye-tracking for detecting specific markers associated with dyscalculia, differentiating them from control groups (Van Viersen et al., 2013). Similarly, technology has demonstrated promise in the early detection and intervention of dysgraphia. Dysgraphia is often diagnosed belatedly since its identification typically occurs only after handwriting skills are fully developed (Van Viersen et al., 2013). Technological tools could therefore support schools in the early screening of this SLD by analyzing indicators such as visual perception skills and eye movements (Van Viersen et al., 2013). These tools provide opportunities to strengthen impaired abilities at an earlier stage (Van Viersen et al., 2013). Piazzalunga et al. (2023) investigated the role of visual perception games and eye-tracking in children with dysgraphia, finding increased inattentiveness and indecisiveness during handwriting tasks. Their study demonstrated that these tools can reasonably predict handwriting difficulties and associated risks, even before children fully develop writing skills (Piazzalunga et al., 2023). In conclusion, these tools show strong potential for early screening of dysgraphia and offer a cost-effective solution with significant benefits (Piazzalunga et al., 2023). Additionally, they could serve as a method to explore the role of specific abilities in the development of handwriting (Piazzalunga et al., 2023). Despite their promise, the current levels of accuracy and precision remain insufficient (Piazzalunga et al., 2023). Longitudinal studies are essential to improve the robustness and generalizability of the findings, paving the way for more reliable and impactful interventions that address fine motor and visual-spatial processing deficits (Piazzalunga et al., 2023; UCL, 2013).

### 7.6 General reading difficulties

General studies on reading difficulties (2/37, 5.4%) highlight that patterns and trends can be identified in players’ results when engaging with educational video games. Research employing serious games, eye-tracking, and clustering techniques has demonstrated their potential in analyzing and enhancing reading performance, with educational profiles emerging from broader assessments (UCL, 2013). Niemelä (2020) applied clustering algorithms to GraphoLearn game log data, identifying six distinct learner profiles among over 1,600 children. These profiles, characterized by specific errors such as confusion between phonetically or visually similar letters, showcase the potential of serious games like GraphoLearn in supporting reading acquisition within transparent writing systems (Niemela, 2020). Learners with higher initial error rates displayed notable progress, proving the game’s effectiveness for severe reading difficulties (Niemela, 2020). While the method enables early identification of children at risk for reading disabilities and supports targeted, personalized interventions, its reliance on early-stage data limits insights into long-term skill development (Niemela, 2020). Future research should focus on expanding datasets, analyzing variables such as syllables and words, and refining clustering algorithms to deepen understanding and enhance game design (Niemela, 2020). Additionally, Peters et al. (2020) investigated the relationship between rapid automatized naming (RAN) and reading skills, demonstrating that fixation metrics from eye-tracking can predict RAN performance and distinguish between children with and without reading difficulties. This highlights the potential of eye-tracking in identifying early indicators of reading challenges, facilitating earlier and more effective interventions (Peters et al., 2020).

These findings underscore the importance of integrating serious games and technology such as eye tracking to personalize educational interventions and identify those at possibly affected by learning disorders early.

### 7.7 Technologies and techniques

Across all the studies that were examined, the most common technology applied was eye-tracking in 21 (56.75%), followed by educational/serious games in 14 studies (37.38%). Machine learning (ML) techniques were identified in 7 studies (18.9%), most often in combination with other equipment like eye-tracking or gamified systems. The focus of this type of research was primarily on ADHD (14 studies) and dyslexia (18 studies), and to a lesser extent on dyscalculia, dysgraphia, or other neurological disorders. Furthermore Eye-tracking was the most generic technology used, most importantly for early identification, as it could track fixations without subject control. Additionally, game-based technologies were used extensively for screening purposes and cognitive training, generally boosting motivation and motivation in children. Moreover, ML-based technologies stepped in to recognize predictive models with high precision in diagnosing neurodevelopmental disorders. Overall, Table 2 reflects a transdisciplinary tendency for integrating interactive environments (e.g., VR, educational games) with computational smartness (e.g., ML, biometric sensing) to deal with the multidimensionality of children’s learning and attention disorders.

### 7.8 Quality appraisal

The MMAT quality analysis results are shown in the table in Supplementary Material 3. A total of 36 studies were included in the review, all of which had identifiable and specific research questions aligned with the available data. Among these, two studies had unclear details regarding the population, and none provided adequate control for confounders due to missing or inadequate follow-up outcomes. This limitation may affect the correct interpretation of the data and introduce potential bias. Additionally, one randomized controlled trial was included, but it remains unclear whether the two groups were comparable at the beginning of the study, whether blinding was applied to the assessor, and whether participants adhered to the intervention.

## 8 Discussions

### 8.1 Principal findings and comparison to prior work

This review underscores the significant advancements in leveraging innovative technologies to address SLDs such as dyslexia, ADHD, dyscalculia, dysgraphia, and general reading difficulties (Vashishtha, 2021; Forbes, 2023). Across these domains, tools like eye-tracking, educational games, and machine learning have emerged as powerful instruments for enhancing diagnosis, understanding, and intervention (Braithwaite et al., 2020; Nazarova et al., 2022; Stokes et al., 2022; Vashishtha, 2021; Forbes, 2023).

Before delving into the principal findings, it is worth clarifying a few points. Even though the beginnings of eye-tracking research were mostly related to the studies of autism spectrum disorder (ASD), this was not part of our inclusion criteria. Therefore, ASD studies were ruled out to sustain a consistent focus on cognitive-academic functions as opposed to social-communication domains (Falck-Ytter et al., 2013).

Moreover, it is noteworthy that multiple prescription-based or regulations-approved therapeutic only games, including EndeavorRx, were returned by our database and registry searches. However, these studies were mainly therapeutic efficacy studies and did not meet the inclusion criteria of this review because they did not either solely or also focus on screening, monitoring or diagnostic performance. Since the aim of this systematic review was to assess digital technologies with the potential to present measurable diagnostic or cognitive indicators, this kind of studies was excluded. However, EndeavorRx continues to be an example of a proven digital therapeutic that points to the growing influence of gamified systems in ADHD management and indirectly reinforces the direction of research discussed in this review (Canadas and Jina, 2022).

Dyslexia was the most extensively studied disorder, with eye-tracking proving particularly transformative (Verwimp et al., 2024; Shultz, 2008). This technology provided critical insights into reading behaviors and oculomotor patterns, offering high diagnostic accuracy and deepening understanding of the reading challenges faced by individuals with dyslexia (Reis et al., 2020). The integration of machine learning with eye-tracking has shown promise for scalable diagnostic tools, while text configuration adjustments and color-based analyses have highlighted opportunities for tailored interventions (FragaGonzlez et al., 2018; Mather and Schneider, 2023; Khaleghi et al., 2022; Reis et al., 2020). Educational games also emerged as engaging tools for both diagnosis and therapy, demonstrating their capacity to address cognitive deficits and enhance reading fluency through gamified experiences (Shultz, 2008).

A similar range of technologies was used for ADHD (Slobodin et al., 2020). Video games offered an engaging medium for assessing cognitive abilities and attention, while also showing therapeutic benefits (Stokes et al., 2022; Garcia-Zapirain et al., 2017; Schmitt et al., 2018). Machine learning approaches demonstrated high accuracy in diagnosing ADHD subtypes, enhancing precision and paving the way for personalized interventions thanks to clustering techniques (Heller et al., 2013; Slobodin et al., 2020; Bishop, 2016). Eye-tracking studies revealed characteristic oculomotor behaviors, providing valuable insights into distractibility and attentional deficits typical of individuals with ADHD (Jafarlou et al., 2017). Combining eye-tracking with additional input sources, such as hand gestures, sensibly enhance the accuracy of the prediction suggesting for a multimodal approach (Rauschenberger et al., 2022). However, increasing the complexity of the tools used subsequently, decreased the accessibility.

Although less frequently studied, dyscalculia and dysgraphia have shown benefit from emerging technologies (Castaldi et al., 2020; Reddy and Reddy, 2016; Van Viersen et al., 2013). Eye-tracking was effective in identifying spatial and numerical representation deficits in dyscalculia, while tools analyzing visual perception and handwriting behaviors showed promise for early dysgraphia screening (Van Viersen et al., 2013). These findings highlight the potential of technology to address challenges in mathematical and writing skills at an earlier stage (Castaldi et al., 2020).

### 8.2 Strengths and limitations

For general reading difficulties, serious games and eye-tracking demonstrated their value in identifying reading patterns and supporting personalized interventions, showing a great potential in tasks like clustering and prediction (Snowling and Hulme, 2012; Korneev et al., 2018; Peters et al., 2020; NICHD, 2020; Snowling and Hulme, 2012). Tools that combined gamified learning with advanced data analysis helped profile learners and provided targeted support, emphasizing the role of technology in early detection and tailored educational strategies (Castaldi et al., 2020; Aro et al., 2019; Karande et al., 2019; Roberts et al., 2012; van de Ven et al., 2017; Milano et al., 2023).

New scientific investigations strengthen the importance of eye-tracking in combination with AI-based innovations for ADHD assessment. Researchers demonstrated the ability of virtual reality in combination with portable eye trackers and 3D games to assess attention processes accurately according to multiple studies (Schmitt et al., 2018; Peters et al., 2020; Niemela, 2020; UCL, 2013; Vashishtha, 2021; Forbes, 2023). The combination of multimodal systems with machine learning algorithms has achieved strong diagnostic success and practical implementations in clinical as well as school environments subsequently emphasizing the significance of these technologies for personalized scalable intervention programs.

Furthermore, from the methodological point of view, this study has several limitations. Firstly, the search strategy was confined to Scopus, PUBMED and clinicaltrials.gov, which might have excluded some evidence available in other databases (e.g., Web of Science). Nevertheless, Scopus, PUBMED and clinicaltrials.gov are among the largest repositories currently available, and we deemed them adequate for our objectives. Secondly, only articles written in English were included, potentially omitting some relevant information. However, non-English articles constituted a negligible proportion of the total. Moreover, although one of our inclusion criteria specified studies involving children up to the age of 18, we occasionally deemed it relevant to include studies that extended participant recruitment to individuals up to the age of 21, even if they primarily focused on children up to 18 [e.g., (Takahashi et al., 2024)]. Finally, the potential for publication bias cannot be overlooked, as it affects all reviews and relies on published literature, which may not reflect all conducted research. Our quality assessment analysis, conducted using the MMAT tool, provides additional insights into the quality of the studies included.

## 9 Implications for future research

Eye-tracking and machine learning have demonstrated remarkable accuracy in controlled environments, but broader validation using larger datasets and more diverse populations is necessary (Takahashi et al., 2024; Fernández-Martín et al., 2024; Yoo et al., 2024; Gomolka et al., 2024; Oh et al., 2024; Toki et al., 2024). Future research should prioritize integrating these technologies into comprehensive frameworks that combine diagnostic precision with tailored interventions. This approach would empower educators and clinicians to more effectively support individuals with SLDs. In addition, it is noteworthy that there is a lack of robust longitudinal studies, which are essential to observe the progression of at-risk groups over time, from assessment to diagnosis. This would help highlight how early intervention improves children’s skills and quality of life over time and identify replicable patterns for early intervention strategies. The road to early screening and intervention for learning disorders is long, but the key findings of this review highlight possible solution paradigms, which can be used to streamline the existing processes and move towards an ever more digital approach, making the process quicker and easier to attain. After all, this is one of the core aims of Paperbox Health, which is looking after the design and validation of serious gaming software aimed at the early diagnosis of neurodevelopmental disorders, leveraging consumer-grade electronics (e.g., smartphones and tablets) and artificial intelligence.

## 10 Conclusion

The findings of this review underscore the significant potential of emerging technologies to transform the diagnosis, understanding, and treatment of SLDs. Nonetheless, critical challenges remain, including scalability, generalizability, and the long-term impact of these innovations, which warrant further exploration. Despite the presence in the scientific literature of this cutting-edge research, little to none has yet been translated into clinical practice. Speech therapists and specialists of neurodevelopmental disorders still recur to paper-based tests and examinations, which are not very efficient in terms of constant monitoring and vary significantly from country to country. With Health 4.0 under way, and Health 5.0 being the future step, digitization and personalization are key pillars for healthcare (Mbunge et al., 2021). This is well-aligned with the UK NHS Long Term Plan aims of improving access to services, enhancing patient experience, supporting clinical decision-making, and enabling more efficient and effective delivery of care (Vashishtha, 2021; NHS, 2019).

## Data Availability

The original contributions presented in the study are included in the article/Supplementary Material, further inquiries can be directed to the corresponding author.

## Supplementary material

SUPPLEMENTARY MATERIAL 1 PRISMA Checklist.

SUPPLEMENTARY MATERIAL 2 A full list of search strings used on different databases.

SUPPLEMENTARY MATERIAL 3 MMAT Quality Analysis Table.
