# Supplementary material for: Serious gaming and eye-tracking for the screening, monitoring, and diagnosis of neurodevelopmental disorders in children: a systematic literature review
Source: Front Bioeng Biotechnol. 2026 Jan 14;13:1672718. doi: 10.3389/fbioe.2025.1672718 (PMC12847422; doi:10.3389/fbioe.2025.1672718)
Supplement: Supplementary file 2 [file Supplementaryfile2.docx]

SCOPUS:

( TITLE-ABS-KEY ( ( "Dyscalculia" OR "dyslexia" OR "reading disorder" OR "learning disorder" OR "dysgraphia" OR "ADHD" OR "neurodevelopmental disorder" OR "executive functions" OR "writing skills" OR "reading skills" ) ) AND TITLE-ABS-KEY ( ( "videogame" OR "machine learning" OR "ML" OR "AI" OR "artificial intelligence" OR "Eye" OR "Video games" OR "Educational games" OR "Machine learning" OR "Vocal analysis" OR "Deep learning" OR "Reinforcement learning" OR "Bayesian estimation" OR "Applied gaming" OR "Clustering" OR "Serious gaming" ) ) AND TITLE-ABS-KEY ( ( "child" OR "children" OR "school" OR "infant" OR "pediatric" OR "preschool" ) ) AND TITLE-ABS-KEY ( ( "screening" OR "monitoring" OR "diagnosis" OR "clinical decision support" OR "assessment" OR "epidemiology" OR "test" OR "testing" OR "therapy" ) ) )

PUBMED:

(((("Dyscalculia"[MeSH] OR "Dyslexia"[MeSH] OR "Learning Disabilities"[MeSH] OR "Agraphia"[MeSH] OR "Attention Deficit Disorder with Hyperactivity"[MeSH] OR "Neurodevelopmental Disorders"[MeSH] OR "Executive Function"[MeSH] OR "writing skills"[Title/Abstract] OR "reading skills"[Title/Abstract]))

AND (("videogame"[Title/Abstract] OR "Video Games"[MeSH] OR "machine learning"[Title/Abstract] OR "ML"[Title/Abstract] OR "AI"[Title/Abstract] OR "artificial intelligence"[Title/Abstract] OR "Eye"[Title/Abstract] OR "Educational games"[Title/Abstract] OR "Machine learning"[Title/Abstract] OR "Vocal analysis"[Title/Abstract] OR "Deep learning"[Title/Abstract] OR "Reinforcement learning"[Title/Abstract] OR "Bayesian estimation"[Title/Abstract] OR "Applied gaming"[Title/Abstract] OR "Clustering"[Title/Abstract] OR "Serious gaming"[Title/Abstract])))

AND (("child"[Title/Abstract] OR "children"[Title/Abstract] OR "school"[Title/Abstract] OR "infant"[Title/Abstract] OR "pediatric"[Title/Abstract] OR "preschool"[Title/Abstract])))

AND (("screening"[Title/Abstract] OR "monitoring"[Title/Abstract] OR "diagnosis"[Title/Abstract] OR "Decision Support Systems, Clinical"[MeSH] OR "assessment"[Title/Abstract] OR "epidemiology"[Title/Abstract] OR "test"[Title/Abstract] OR "testing"[Title/Abstract] OR "therapy"[Title/Abstract]))))
